# Supplementary material for: A sensation for inflation: initial swim bladder inflation in larval zebrafish is mediated by the mechanosensory lateral line
Source: J Exp Biol. 2023 Jun 5;226(11):jeb245635. doi: 10.1242/jeb.245635 (PMC10281257; doi:10.1242/jeb.245635)
Supplement: Supplementary information [file jexbio-226-245635-s1.pdf]

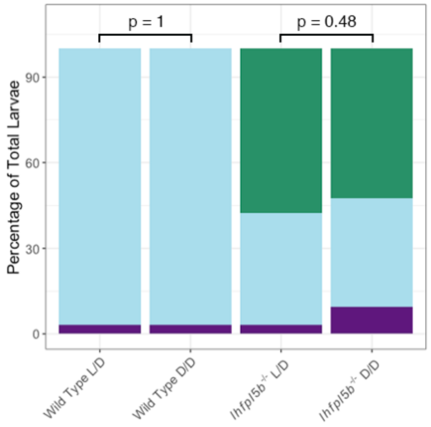

Fig. S1. Buoyancy proportions of larvae raised in total darkness. Percentage of larvae that exhibit swim bladder under, regular, and over-inflation raised in either a 14:10 light/dark cycle (L/D) or total darkness (D/D). A Chi-Squared Test was used to assess significance. Both Wild Type L/D and Wild Type D/D over-inflation proportions: M = 0.0%, SD = 0.0%, n = 32. *lhfpl5b*$^{-/-}$ L/D over-inflation proportion: M = 57.5%, SD = 6.6%, n = 30. *lhfpl5b*$^{-/-}$ D/D over-inflation proportion: M = 52.4%, SD = 4.1%, n = 30.

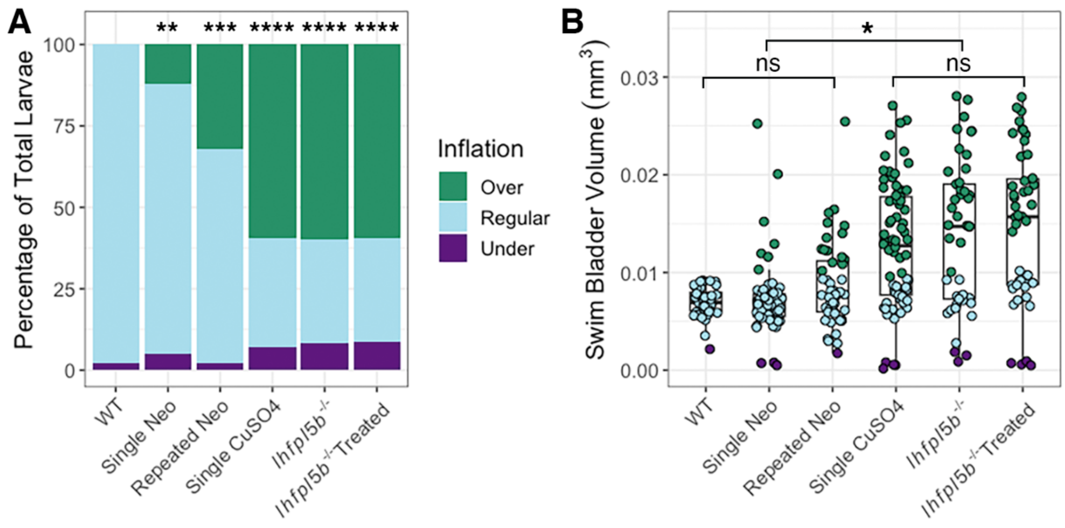

Fig. S2. Ototoxic ablations of the lateral line. A) Percentage of larvae that exhibit swim bladder under, regular, and over-inflation. A Chi-Squared Test was used to assess significance. B) Swim bladder volume (mm^3) in 5 dpf larvae. A One-Way ANOVA was used to determine significance. **** = $p < 0.0001$, *** = $p < 0.001$, ** = $p < 0.01$, ns = no significance. Complete ANOVA and Tukey statistics are available in Data S1.

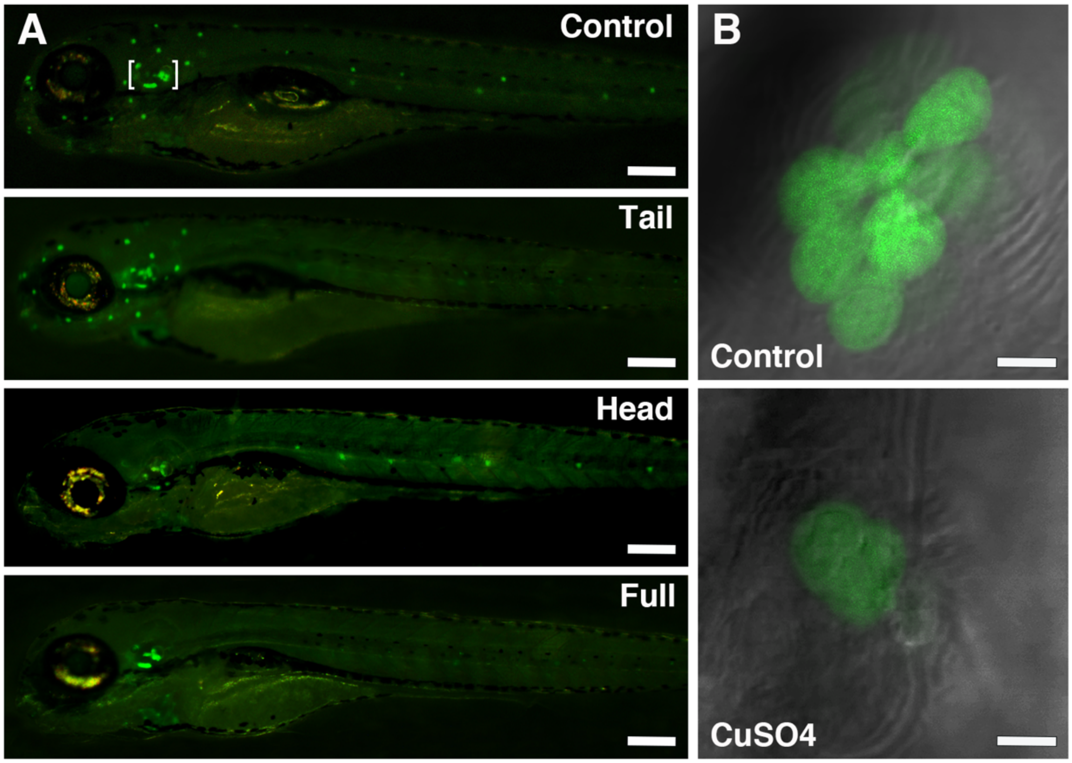

Fig. S3. Anterior and posterior-specific lateral line ablations with CuSO4. A) Representative images of live *Tg(myo6b:EGFP)vo68* transgenics at 4 dpf, two hours after CuSO4 treatment. This transgene labels hair cells of the lateral line and inner ear (indicated by white brackets on the Control image), and treatment only affects lateral line hair cells so inner ear expression remains post-treatment. Scale bars = 0.2 mm. B) Representative images from untreated Control and CuSO4-treated L1 neuromasts. Scale bars = 5 μM.

Journal of Experimental Biology • Supplementary information

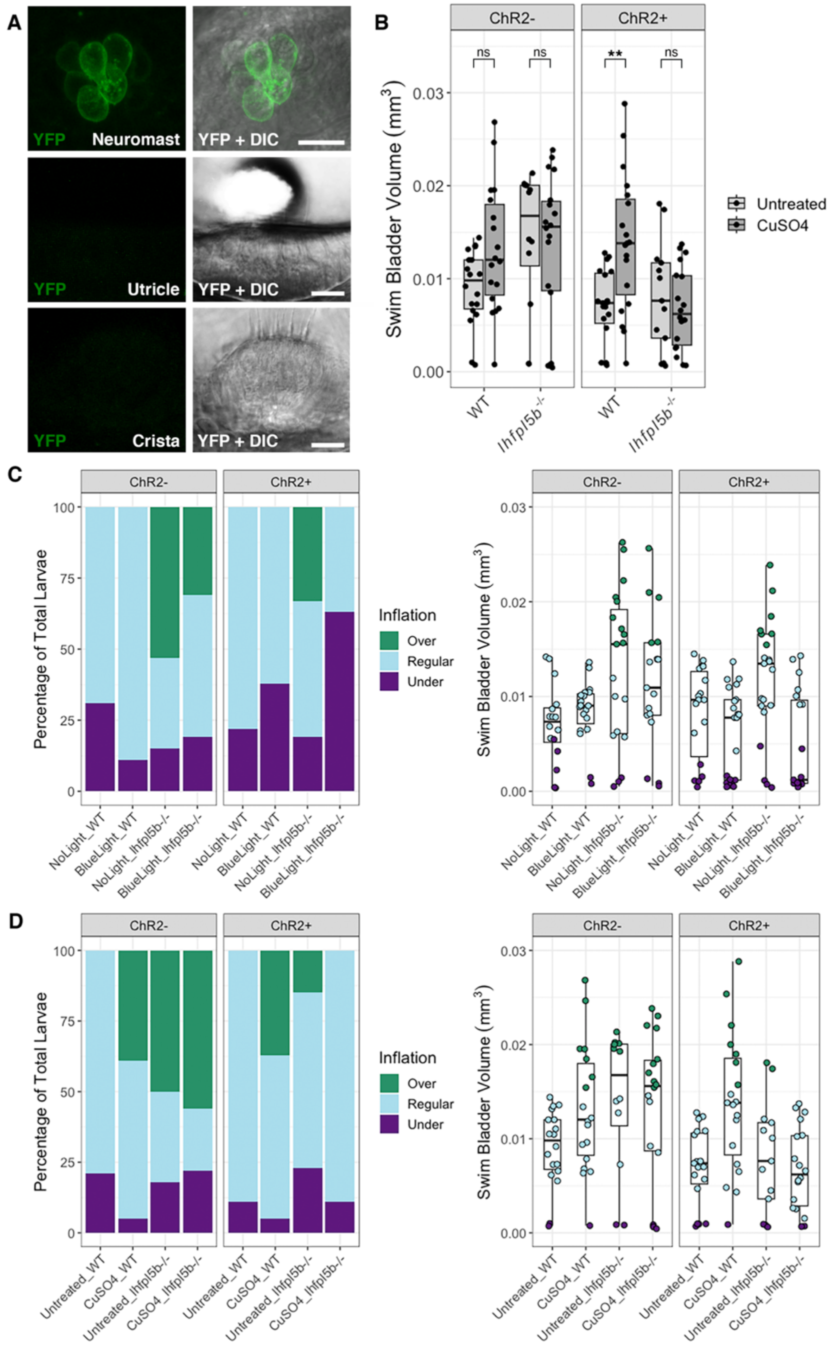

Fig. S4. Channelrhodopsin-2 (ChR2) expression in *Tg(lhfpl5b2028:ChR2-EYFP-pA)* transgenics.

A) Images of ChR2-YFP in hair cells in a 2 dpf neuromast, utricle, and crista where fluorescence expression is only seen in the neuromasts of the lateral line. First panel is YFP only (false-colored green) and the second panel is a merge of YFP and DIC. Scale bars = 10 uM. B) Swim bladder volume (mm^3) of 6 dpf larvae from the experimental condition (blue light overhead). A One-Way ANOVA was used to determine significance. ** = $p < 0.00724$, ns = no significance. Complete ANOVA and Tukey statistics are available in Data S1. C) Percentages and box plots of experiment in Fig. 6 to show inflation categories. D) Percentages and box plots of experiment in Fig. S4B to show inflation categories.

Table S1.

Click here to download Table S1

Journal of Experimental Biology • Supplementary information
